# PCR-DGGE Analysis: Unravelling Complex Mixtures of Badnavirus Sequences Present in Yam Germplasm

**DOI:** 10.3390/v9070181

**Published:** 2017-07-11

**Authors:** Aliyu A. Turaki, Moritz Bömer, Gonçalo Silva, P. Lava Kumar, Susan E. Seal

**Affiliations:** 1 Natural Resources Institute, University of Greenwich, Central Avenue, Chatham Maritime, Kent ME4 4TB, UK; a.turaki@agshare.today (A.A.T.); G.Silva@greenwich.ac.uk (G.S.); S.E.Seal@greenwich.ac.uk (S.E.S.); 2 Kebbi State University of Science and Technology Aliero, Sokoto Road, PMB 1144 Birnin Kebbi, Nigeria; 3 International Institute of Tropical Agriculture (IITA), Oyo Road, PMB 5320 Ibadan, Nigeria; L.Kumar@cgiar.org

**Keywords:** Badnavirus, denaturing gradient gel electrophoresis (DGGE), sequence diversity, yam, *Dioscorea* spp., endogenous pararetrovirus (EPRV), episomal badnavirus, integration, detection

## Abstract

Badnaviruses (family *Caulimoviridae*, genus *Badnavirus*) have emerged as serious pathogens especially affecting the cultivation of tropical crops. Badnavirus sequences can be integrated in host genomes, complicating the detection of episomal infections and the assessment of viral genetic diversity in samples containing a complex mixture of sequences. Yam (*Dioscorea* spp.) plants are hosts to a diverse range of badnavirus species, and recent findings have suggested that mixed infections occur frequently in West African yam germplasm. Historically, the determination of the diversity of badnaviruses present in yam breeding lines has been achieved by cloning and sequencing of polymerase chain reaction (PCR) products. In this study, the molecular diversity of partial reverse transcriptase (RT)-ribonuclease H (RNaseH) sequences from yam badnaviruses was analysed using PCR-dependent denaturing gradient gel electrophoresis (PCR-DGGE). This resulted in the identification of complex ‘fingerprints’ composed of multiple sequences of *Dioscorea* bacilliform viruses (DBVs). Many of these sequences show high nucleotide identities to endogenous DBV (eDBV) sequences deposited in GenBank, and fall into six monophyletic species groups. Our findings highlight PCR-DGGE as a powerful tool in badnavirus diversity studies enabling a rapid indication of sequence diversity as well as potential candidate integrated sequences revealed by their conserved nature across germplasm.

## 1. Introduction

Badnaviruses are plant pararetroviruses (family *Caulimoviridae*, genus *Badnavirus*) infecting a broad range of economically important crop plants and have emerged as serious pathogens especially affecting the cultivation of tropical crops, such as banana, black pepper, cacao, citrus, sugarcane, taro and yam [1]. The development of reliable diagnostic tests for badnaviruses is challenging due to high serological and genetic heterogeneity, as experienced in a wide range of crops [2,3,4,5]. The discovery of integrated badnavirus sequences in some host plant genomes of agricultural and horticultural crops complicates the fool-proof diagnosis of episomal infections using nucleic acid-based methods as experienced for banana streak viruses (BSVs) in *Musa* spp. [6,7,8], and their existence poses further challenges for taxonomy, safe movement of germplasm, and disease management (reviewed by [9]).

Integrated sequences derived from representatives of four out of eight recognized genera of the *Caulimoviridae* family have been found in 27 species from nine different plant families according to Geering et al. [10]. Termed endogenous pararetroviruses (EPRVs) [11,12,13], integration events have been most studied in banana [14], petunia [15], tobacco [16], rice [17], potato [18] and tomato [19], and the majority of these integrated sequences described so far are fragmented and rearranged [7,20,21,22]. Few of these EPRVs can be activated to give rise to infective episomal forms initiating systemic badnavirus infections de novo [20,22,23]. Four species of the genus *Badnavirus* described in banana genomes of *Musa balbisiana* species, namely *Banana streak OL virus* (BSOLV), *Banana streak IM virus* (BSIMV), *Banana streak MY virus* (BSMYV), and *Banana streak GF virus* (BSGFV) [21,24,25,26], as well as the petuvirus *Petunia vein clearing virus* (PVCV) in petunia [22], and the solendovirus *Tobacco vein-clearing virus* (TVCV) in tobacco [16,23] are the only activatable EPRVs discovered to date. Activation is considered in banana to be triggered by tissue culture, hybridization, or temperature differences in newly created banana interspecific hybrids [27,28,29], and a homologous recombination-based model is proposed to explain the release of the BSOLV genome from its endogenous counterpart [30].

Increasing numbers of integrated viral sequences are now identified in next-generation sequencing (NGS) data. This for example includes the recent discovery of the endogenous florendoviruses (family *Caulimoviridae*, genus *Florendovirus*) as major components of a large diversity of flowering plant genomes, such as apple, cacao, cassava, citrus, grape, maize, papaya, potato, rice and tomato among others [10]. The analysis of these endogenous sequences is substantially more difficult in plant species still lacking a good quality reference genome as is the case for some staple crops, in particular tropical root and tuber crops. The nature of EPRV sequences can be of concern to breeding programmes and multiplication processes with the goal of distributing large amounts of clean vegetative propagation material. Germplasm-containing activatable EPRVs would need to be removed from such programmes prior to multiplication. In this study, we illustrate the value of PCR-dependent denaturing gradient gel electrophoresis (PCR-DGGE) analysis in unravelling complex mixtures of badnavirus sequences through the example of yam germplasm.

Yam (*Dioscorea* spp.) is one of the most important staple food crops, playing a major role in food security in West Africa [31,32]. *Dioscorea* bacilliform viruses (DBVs) (family *Caulimoviridae*, genus *Badnavirus*) contain several members that are a concern to the safe movement of the germplasm of this vegetatively-propagated crop [3,33,34]. Sequences representative of the genus *Badnavirus* have been shown to be prevalent in yam materials tested from all yam growing areas of the world, and are highly heterogeneous [3,34,35,36,37,38]. Integrated badnavirus sequences have been discovered in the genomes of Guinea yam (*Dioscorea cayenensis-rotundata* complex), termed endogenous DBV (eDBV) sequences [38,39]. The rapid increase in the discovery of new plant pararetroviruses and their integrated sequences over recent years has been the result of improved PCR diagnostics and an increase in research attention [3,21,40,41,42]. Understanding the complexity of badnavirus sequences in plant germplasm is of great importance to virologists and plant breeders, in particular when working with vegetatively-propagated crops with an urgent need for a sustainable supply of virus-free planting material. A pressing need remains in the development of diagnostic tools to differentiate the integrated sequences from those representing episomal particles, such that the potential distribution of virus infections can be assessed and their impact on crop yields determined.

Denaturing gradient gel electrophoresis (DGGE) is a commonly used molecular technique for rapid fingerprint analysis that was first described by Fischer and Lerman [43]. DGGE is capable of separating double-stranded PCR products of similar length but differing sequence composition (reviewed by [44]). The principle of the technique is based on the physicochemical fundamental of DNA base-pairing and the differing mobility of double-stranded and partially denatured DNA when analysed by polyacrylamide gel electrophoresis [44,45]. The addition of a GC clamp to the 5′ end of the primer sequence prevents complete denaturation of DNA molecules during DGGE analysis. The technique detects variation of small DNA fragments (~200–700 bp) that differ by as little as a single base substitution [46,47,48]. DGGE has been used extensively for diversity studies such as microbial biodiversity [45,49,50,51], fungal communities [52], genomes of viral strains [53,54,55], forensic application [56] and plant genome polymorphisms [57,58].

The aim of this study was to determine: (1) whether PCR-DGGE could be used as a rapid technique to screen for differences in complex mixtures of episomal and endogenous badnavirus sequences being present in West African yam landraces and breeding lines; and (2) if this approach would enable the simple identification of integrated badnavirus sequences conserved across germplasm. Historically, this was achieved by cloning and sequencing of PCR products using the generic badnavirus primer pair Badna-forward primer/reverse primer (FP/-RP) [59]. This approach however has the disadvantage of there being a possibility of failing to identify all sequence diversity due to clones selected not being representative of the total diversity present within a tested plant. Restriction fragment length polymorphism (RFLP) analysis has also been used to study yam badnavirus diversity [3,35,60] but this technique shows a lower resolution of diversity than DGGE. However, neither technique (cloning of PCR products and RFLP) allows episomal and endogenous badnavirus sequences to be distinguished.

**Figure 1:**
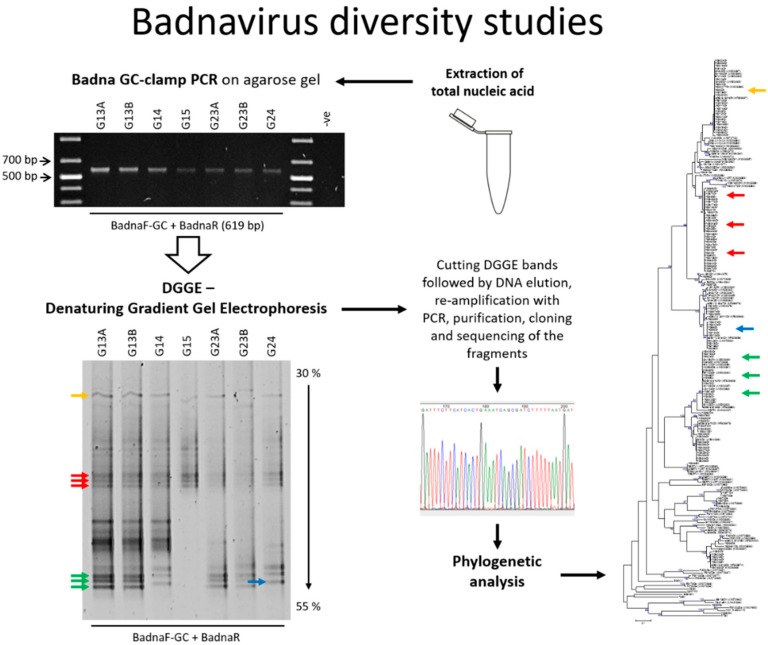
Graphical workflow describing badnavirus diversity studies using denaturing gradient gel electrophoresis (DGGE). PCR: polymerase chain reaction.

This study is the first to demonstrate the value of PCR-DGGE (hereafter referred to as DGGE) for unravelling complex sequence mixtures present in badnavirus PCR products amplified from yam breeding lines (Figure 1). This approach resulted in the generation of 114 nucleotide sequences from yam germplasm which have been deposited in the GenBank database under accession numbers KY555456 to KY555569. These DBV sequences fall into six monophyletic species groups and represent several unique DGGE profiles. Interestingly, many bands are conserved across germplasm indicating integrated sequences, and many sequences identified in this study do show high nucleotide identities to eDBV sequences. It is considered that the ability to link DGGE profiles to badnavirus sequences will assist the future identification of badnavirus diversity based on comparing sample profiles to standard profile markers.

## 2. Materials and Methods

### 2.1. Plant Material

Yam leaf samples of breeding lines and landraces (47 samples in total) of *Dioscorea alata* (*n* = 11), *Dioscorea bulbifera* (*n* = 1), *Dioscorea cayenensis* (*n* = 1), *Dioscorea dumetorum* (*n* = 3), *Dioscorea esculenta* (*n* = 2) and *Dioscorea rotundata* (*n* = 23) used in this study were collected from screen-houses at the International Institute of Tropical Agriculture (IITA, Ibadan, Nigeria) and placed in polythene bags (14 cm × 14 cm). The breeding lines were a subset of the IITA collection samples (*n* = 127) consisting of first filial (F^1^) generations of West African breeding lines of *D. rotundata* (*n* = 112) and *D. rotundata* landraces (*n* = 15). Details of samples from these collections are given in the Supplementary materials of Seal et al. [38]. Selected yam breeding lines and landraces of *D. rotundata* (*n* = 6) were provided by the IITA and tubers were grown in a quarantine aphid-proof glasshouse at the Natural Resources Institute (NRI, Chatham Maritime, UK), as described by Mumford and Seal [61]. Individual leaf samples were collected from each plant in small polythene bags (10 cm × 15 cm) and processed immediately. *Dioscorea rotundata* accession (TDr) 89/02475A and B, and TDr 1892A and B are clones of the same yam accessions.

### 2.2. Total Nucleic Acid Extraction from Yam Leaves and PCR Amplification of Badnavirus Sequences

Total nucleic acids were extracted from fresh yam leaf tissue (~100 mg) using a modified cetyltrimethylammonium bromide (CTAB) method as described by Kenyon et al. [3]. Total nucleic acids were screened for the presence of sequences typical of the genus *Badnavirus* by PCR using the degenerate primer set Badna-forward primer (FP) and Badna-reverse primer (RP) designed by Yang et al. [59]. These Badna-FP/-RP primers amplify a 579-bp region (528 bp excluding primer sequences and representing only complete amino acids) of the reverse transcriptase (RT)-ribonuclease H (RNaseH) gene used for taxonomic assessment of badnaviruses [62]. To confirm the suitability of DNA for PCR amplification all DNA samples were first screened using primers targeting the yam actin gene as described by Silva et al. [63]. PCR amplifications were set up in 25-µL reactions containing 1 µL of template (20 ng), 0.5 µM of each primer, 0.25 mM of each deoxynucleotide triphosphate (dNTP), 1 U DreamTaq DNA polymerase and 1× DreamTaq Green buffer (Thermo Scientific, Loughborough, UK) containing 2 mM MgCl_2_. The cycle conditions for PCR amplification were 94 °C for 2 min, followed by 40 cycles of 94 °C for 20 s, 55 °C for 30 s, 72 °C for 1 min and a final extension of 72 °C for 10 min. PCR products were analysed by gel electrophoresis through 1.5% (*w/v*) agarose gels including 1× RedSafe nucleic acid stain (iNtRON Biotechnology, Gyeonggi-do, Korea) in 0.5× Tris-Boric acid-ethylenediaminetetra acetic acid (EDTA) (TBE) buffer. PCR products of the expected size were purified and diluted PCR products (‘nested’) or diluted total nucleic acid extractions (‘direct’) were used as template for the purposes of generating PCR products designated for DGGE analysis. Both Badna-FP and Badna-RP primers were modified by the addition of a GC clamp (5′ CGC CCG CCG CGC GCG GCG GGC GGG GCG GGG GCA CGG GGG GAT GCC ITT YGG IIT IAA RAA YGC ICC 3′ and 5′ CGC CCG CCG CGC GCG GCG GGC GGG GCG GGG GCA CGG GGG GCC AYT TRC AIA CIS CIC CCC AIC C 3′, respectively) generating a product of 619 bp. PCR reactions using the Badna-FP/-RP GC-clamp primers used cycle conditions as follows: one cycle at 95 °C for 5 min, then 35 cycles of 94 °C for 1 min, 55 °C for 45 s, 72 °C for 2 min followed by one cycle of extension at 72 °C for 10 min. Prior to DGGE analysis, PCR products were confirmed to be of the correct size by agarose gel electrophoresis. All sequencing in this study was performed by Source BioScience (Nottingham, UK). All primers described were synthesised using Sigma oligo service (Sigma-Aldrich, Irvine, UK) and reSource (Source BioScience, Nottingham, UK), or Sure Clean kit (Bioline, London, UK) purification kits were used to clean PCR products prior to sequencing or cloning.

### 2.3. Denaturing Gradient Gel Electrophoresis (DGGE)

DGGE was performed using the INGENYphorU-2×2 apparatus (INGENY, Goes, The Netherlands) according to the manufacturers’ instructions and following procedural comments provided in the protocol by Green et al. [44]. Gradient gels containing 6.5% (*v/v*) polyacrylamide (37.5:1 ratio of acrylamide:bis-acrylamide) (National Diagnostics, Atlanta, USA) were formed using a peristaltic pump (Rietschle Thomas, Schopfheim, Germany) and a gradient maker device (INGENY) with denaturing gradients from 35 to 50% (top to bottom) unless stated otherwise (where 100% is 7 M urea and 40% (*v/v*) deionized formamide) in 1× Tris-acetate-EDTA (TAE) electrophoresis buffer. Samples (20 µL) were loaded on a stacking gel. Electrophoresis was performed at 80 V at a temperature of 60 °C for 18 h. Gels were stained with 1× SYBR Gold nucleic acid gel stain (Invitrogen, Life Technologies, Paisley, UK) in 1× TAE for 30 min at room temperature and destained in deionized water. Gels were placed on a UV transilluminator (G-box Chemi HR16, Syngene, Cambridge, UK) and visualized. Bands of interest were excised from DGGE gels using a sterile scalpel and DNA eluted by soaking in 100 µL of molecular grade water (Sigma) at 4 °C overnight. Aliquots were diluted 1:10 and re-amplified by PCR using the Badna-FP/-RP primer pair followed by PCR purification. Purified PCR products were cloned prior to sequencing using the pGEM-T Easy vector system (Promega, Southampton, UK) according to the manufacturers’ instructions and standard sequencing primers SP6 and T7. To obtain a consensus sequence and control for cross-contamination, two clones from each excised DGGE band showing a different migration pattern were sequenced.

### 2.4. Sequence Analysis and Phylogeny

Yam badnavirus partial reverse transcriptase-ribonuclease H (RT-RNaseH) nucleotide sequences generated from plasmid clones were analysed using MEGA version 6.0 [64]. The Badna-FP/-RP and vector sequences were removed and the edited sequences were used for similarity basic local alignment search tool (BLAST) searches in the National Centre for Biotechnology Information (NCBI) GenBank databases [65]. Multiple alignments of the partial RT-RNaseH sequences were performed using the CLUSTALW default settings in MEGA version 6.0, where phylogenetic trees were created using the maximum likelihood method with the Kimura 2-parameter model [66]. The robustness of trees was determined by generating bootstrap consensus trees using 1000 replicates. A nucleotide percent similarity matrix (Table S1) was generated using Multiple Alignment using Fast Fourier Transform [67,68]. Protein sequences were aligned using CLUSTAL OMEGA [69,70] and further processed in BioEdit version 7.2.5 [71]. According to the International Committee on Taxonomy of Viruses (ICTV), sequences of the genus *Badnavirus* differing in their partial RT-RNaseH coding region by more than 20% meet the species demarcation criteria [62]. Eighty-nine yam badnavirus partial RT-RNaseH sequences (see Figure 4 for accession numbers) and the following virus sequences were obtained from the GenBank and used for comparative analyses: *Banana streak OL virus* (BSOLV, AJ002234); *Cacao swollen shoot virus* (CSSV, AJ781003); *Commelina yellow mottle virus* (ComYMV, NC001343); *Rice tungro bacilliform virus* (RTBV, X57924); *Sugarcane bacilliform MO virus* (SCBMOV, M89923); and *Taro bacilliform virus* (TaBV, AF357836). For consistency, the grouping system reported by Kenyon et al. [3] was adopted in this study. One new group (U12) reported by Umber et al. [39] and three new groups (T13–T15) described by Bömer et al. [33] were also added to the phylogenetic analysis (Figure 4).

## 3. Results

### 3.1. DGGE Resolves a Complex Mixture of Badnavirus Sequences Present in *Dioscorea* Species

**Figure 2:**
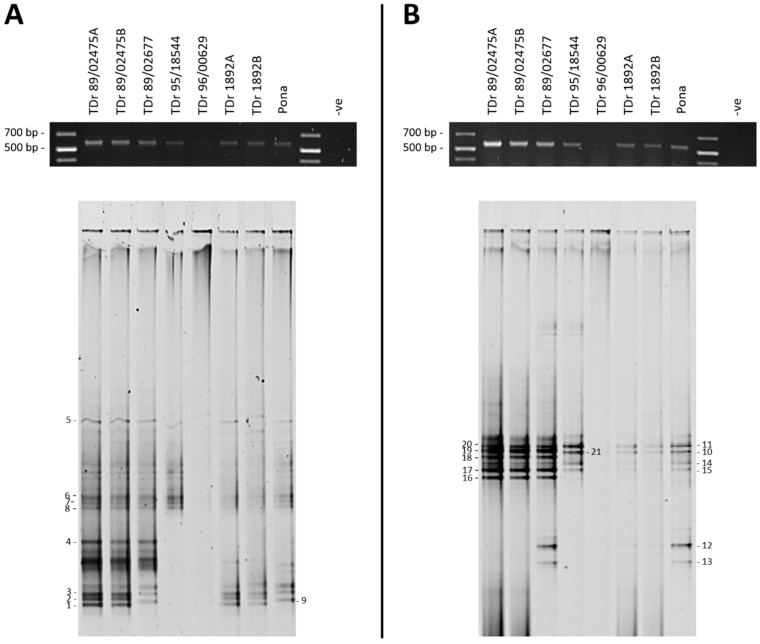
Denaturing gradient gel electrophoresis (DGGE) analysis of partial reverse transcriptase-ribonuclease H (RT-RNaseH) badnavirus sequences from seven *Dioscorea rotundata* breeding lines and one landrace comparing PCR amplifications using the generic badnavirus primer pair Badna-forward primer/reverse primer (FP/-RP) with a GC clamp fused to the forward (**A**) or reverse (**B**) primer. PCR conditions were as outlined in the Materials and Methods section, but using undiluted DNA extractions (‘direct’) and only 25 cycles. PCR amplifications (5 µL) were checked for their expected size (619 bp) on 1% w/v agarose in 0.5× Tris-Boric acid-ethylenediaminetetraacetic acid (EDTA) (TBE) gels (black gels on top) before DGGE loading (20 µL). The denaturing gradient was 30–55% and DGGE was performed at 80 V at a temperature of 60 °C for 18 h. Band numbers 1–21 were excised and cloned. The corresponding sequences are presented in Table 1. *Dioscorea rotundata* accession TDr 89/02475A and B and TDr 1892A and B are clones of the same yam accessions.

To evaluate the potential of DGGE in unravelling the complexity of DBV diversity, yam DNAs (*n* = 47) were selected based on all those scoring PCR-positive for badnavirus sequences using the generic badnavirus primer pair Badna-FP/-RP. Yang et al. [59] designed this degenerate primer pair based on the consensus sequences of RT and RNaseH coding regions of published badnavirus sequences at the time. These primers are widely used in badnavirus research and also proved to be functional in several DBV diversity studies performed by Bousalem et al. [34], Kenyon et al. [3] and Seal et al. [38] among others. DGGE analysis of Badna-FP/-RP positive PCR products required the addition of a GC clamp to one of the two primers. A GC clamp is usually positioned at the 5′ end of the forward primer [44]. Due to the degeneracy of the generic badnavirus primer set, we decided to test the addition of a GC clamp to both, the Badna-forward as well as the—reverse primer. A subset of eight yam samples grown at the NRI’s quarantine glasshouse was selected and the variability of DBV sequences amplified using the two different Badna GC-clamped primers was compared by DGGE. Distinctive bands that were found to be sharp and intense were excised, cloned and sequenced (Figure 2).

Both Badna GC-clamped primers were able to produce PCR amplifications of the expected 619-bp size with only sample TDr 96/00629 failing in both reactions, the probable cause considered to be PCR inhibitors (Figure 2A,B). ‘Direct’ PCR amplification used the GC-clamped primers with DNA extractions as template, rather than ‘nested’ conditions, where purified Badna-FP/-RP products were used as templates and re-amplified. The ‘direct’ approach was found to improve the resolution of the PCR products analysed by DGGE and resulted in distinct but less intense bands on agarose gels when a lower number of PCR cycles (e.g., 25 cycles) was applied. These relatively sharp and intense DGGE bands could be excised under minimal UV exposure. Conversely, very intense PCR bands as a result of more PCR cycles being applied (35–40 cycles), led to an increase in smearing and less distinct banding patterns during DGGE analysis. Twenty-one bands were excised and successfully processed further (Figure 2).

To obtain phylogenetic information from DGGE analysis, the excised bands were re-amplified, cloned and sequenced. This step lowered the likelihood of obtaining multiple DNA sequences from a single band of interest due to the close migration of different sequences on DGGE gels. With the exception of some samples, two clones per excised DGGE band were sequenced, with both clone sequences being presented unless they were found to be 100% identical to each other. The clone sequence data set (Table 1) was coded as follows: the first two letters stand for the country of origin (NG = Nigeria), ‘b’ represents breeding line samples, ‘l’ represents landrace yam samples, the middle number denotes the position of the excised DGGE band, the next letter denotes the clone (a = clone a and b = clone b) and the last two letters refer to the *Dioscorea* host species (e.g., Dr = *Dioscorea rotundata*).

Duplicate clone sequences originating from DGGE band numbers 1–21 resulted in >98% identical sequences, except for band numbers 8, 16 and 18. Only one clone was sequenced successfully for each of the DGGE bands 4 and 5 (Figure 2 and Table 1). Although DGGE bands from different plant samples migrating at the same position in the gel usually resulted in >99% identical sequences (e.g., see bands 10 and 21), sometimes >99% identical sequences migrated at different positions. This was for example the case for bands 12 and 13, that showed 99–100% nucleotide identity to the eDBV 12-clone sequence S1a4Dr (KF829956, [39]) (Table 1). Two of the four clone sequences generated from DGGE bands 12 and 13, NGl12bDr and NGl13aDr are 100% identical across the 528-bp region used for taxonomic assessment of badnaviruses [62]. However, they differ in their primer sequences (determined by examining the cloned excised band sequences), and thus the migration of these sequences at different positions in DGGE is likely a result of the significant degeneracy present in the Badna-FP/-RP primer pair.

The reproducibility of the DGGE technique was tested by running samples from two individual plants for both TDr 89/02475 and TDr 1892 accessions. The DGGE patterns for these biological replicates were found to be identical in both of the primer combinations tested (Figure 2). Nine DGGE bands of PCR amplifications using the Badna-F GC-clamp primer (Figure 2A) included DBV sequences assigned to four different monophyletic groups according to the phylogenetic analysis presented in Figure 4. Sequences clustered into groups K9 (bands 1–3), K8 (all of bands 4–7, one band 8 clone NGb8bDr), U12 (band 9) and T13 (band 8 clone NGb8aDr). Several more bands of low intensity were excised, but could not be processed successfully. The majority of sequences from DGGE bands 10–21 amplified using the Badna-R GC-clamp primer (Figure 1B) clustered into group K8 (Table 1). Band numbers 12 and 13 represent sequences of group U12, showing two distinct positions in the DGGE analysis. The only group K9 sequence was identified for band number 16 (NGb16bDr), however the second clone NGb16aDr represented a K8 sequence. In comparison, these findings suggest that group K9 sequences could be under-represented in PCR amplifications using the Badna-R GC-clamp primer. Hence, we decided to use the Badna-F GC-clamp primer in DGGE analysis hereafter.

Screening of *D. rotundata* breeding lines (*n* = 112) and landraces (*n* = 15) maintained at the IITA generated 100% badnavirus PCR-positive results in a study performed by Seal et al. [38] (details of samples given in Supplementary materials of [38]). In this study, DGGE was used to study the diversity of the badnavirus sequences that exist within a subset of the 100% PCR-positive samples (Table 1). Our DGGE analysis revealed several unique bands but also many bands that are shared in a high proportion of the yam material tested. A total of at least 15 distinct bands were differentiated by DGGE, and following sequencing these could be assigned to different partial RT-RNaseH sequences. The dataset (Table 1) created reflects the complexity of the diversity of badnavirus sequences present in *Dioscorea* species.

**Figure 3:**
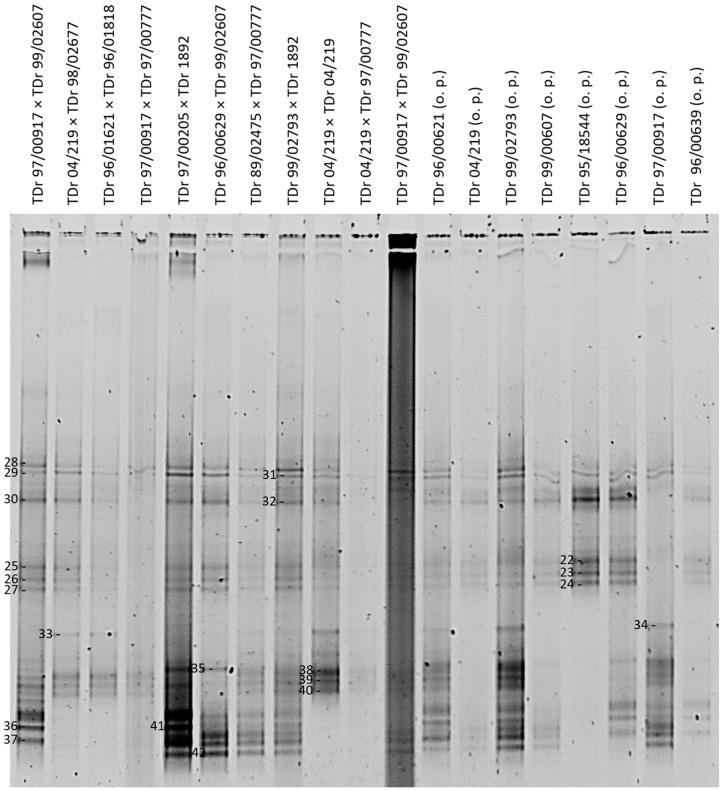
DGGE analysis of partial RT-RNaseH badnavirus sequences from 19 samples consisting of first filial (F^1^) generations of West African breeding lines (o.p. stands for open pollinated) of *D. rotundata* comparing patterns of PCR amplifications (20 µL loaded) using the generic badnavirus primer pair Badna-FP/-RP with a GC clamp fused to the forward primer. The denaturing gradient was 35–50% and DGGE was performed at 80 V at a temperature of 60 °C for 18 h. Band numbers 22–42 were excised and cloned. The corresponding sequences are presented in Table 1.

Twenty-one DGGE bands were successfully cloned and sequenced from the DGGE gel presented in Figure 3. Several sequences (DGGE bands) appear to be common to all breeding lines of *D. rotundata*. This is for example the case for sequences NGl14bDr, NGb30bDr and NGb55aDr, all clustering with species group K8 (Table 1), and being 100% identical to BfA103Dc (AM503393, [34]) and 99% identical to known endogenous sequences of K8, such as S2h9Dr (KF829997, [39]). BLAST similarity matches showing 99–100% identity to BfA103Dc were identified for another 23 DGGE clone sequences (Table 1). Additionally, DGGE patterns can be compared across the samples tested and similarities or differences are easily observed. For example, crossings TDr 89/02475 × TDr 97/00777 and TDr 99/02793 × TDr 1892 showed identical DGGE patterns, suggesting the presence of the same set of DBV sequences.

Eleven cross-breeding lines of *D. alata* were also analysed in this study (Figure S1). Nine out of these 11 cross-breeding lines contain a double-band made out of sequences NGb60Da and NGb61Da, which were found to be 92% identical to GyJT2Dt (AM503389, [34]) and 93% identical to NG1Da (AM944571, [35]), respectively. Sequence NGb60Da appears to be not only common to the majority of *D. alata* breeding lines tested in this study, but is also common to most of the *D. rotundata* material tested (e.g., sequence NGb6aDr corresponding to band 6 in Figure 2). Additionally, eight of the 11 cross-breeding lines analysed contain a second double-band which was not resolved very well, but contained sequence NGb63Da identified as 99% identical to SB42Da (AM072696), which was isolated from a *D. alata* plant in the Solomon Islands in 2000 [34] and fell into monophyletic group K1 (Table 1). Both double-bands appear to be conserved across most of the *D. alata* breeding lines analysed in this study and suggest the presence of integrated sequences in *D. alata* germplasm.

### 3.2. Phylogenetic Diversity of Dioscorea Badnavirus Sequences

**Figure 4:**
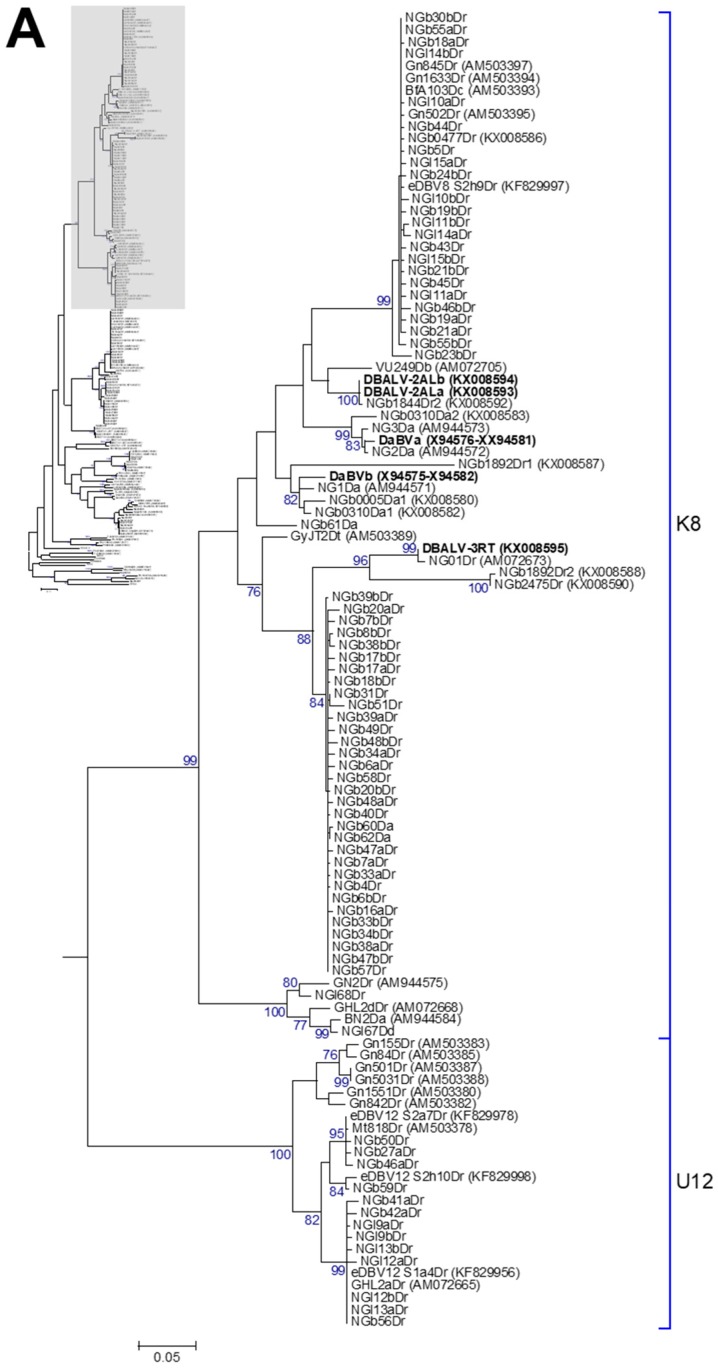

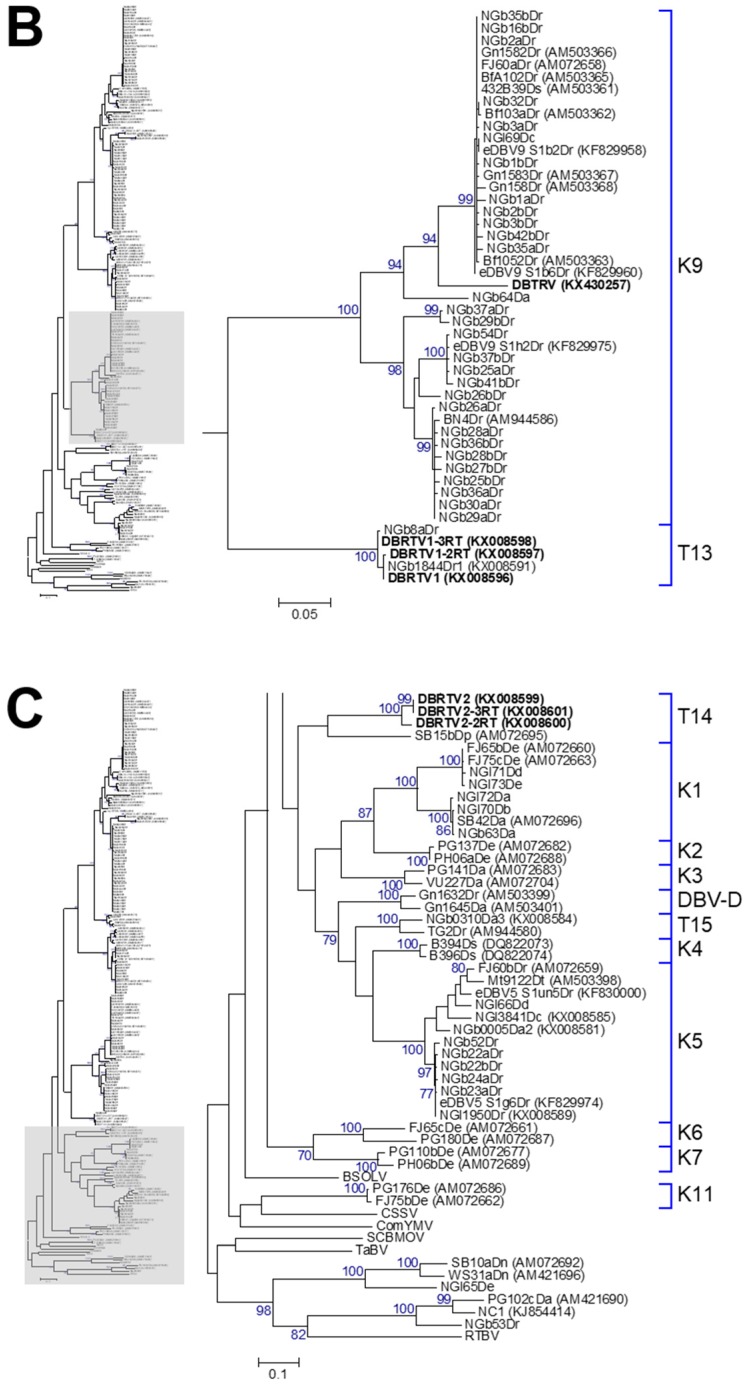
Bootstrap consensus phylogenetic tree using the maximum likelihood method built from the badnavirus 527–528-bp-long partial RT-RNaseH nucleotide sequences of 114 yam badnavirus sequences determined in this study. Included in the analysis are partial RT-RNaseH sequences with names and accession numbers from GenBank of previously analysed yam samples by Bömer et al. [33], Bousalem et al. [34], Eni et al. [35], Kenyon et al. [3], Seal et al. [38] and Umber et al. [39,72]. Equivalent sequences from *Cacao swollen shoot virus* (CSSV, AJ781003), *Banana streak OL virus* (BSOLV, AJ002234), *Commelina yellow mottle virus* (ComYMV, NC001343), *Sugarcane bacilliform MO virus* (SCBMOV, M89923), *Taro bacilliform virus* (TaBV, AF357836) and outgroup *Rice tungro bacilliform virus* (RTBV, X57924) were added, as well as representative sequences of all monophyletic groups described by Bousalem et al. [34] (where DBV-D: Dioscorea bacilliform virus D), by Umber et al. [39] and by Kenyon et al. [3], denoted by U12 and K1–K11 respectively. Three novel monophyletic groups, T13–15, described by Bömer et al. [33] were also included. Sequences depicted in bold represent partial RT-RNaseH sequences of characterised episomal full-length DBV genomes currently available in GenBank. The phylogenetic tree was divided into sub-groups with groups K8 and U12 presented in (**A**); K9 and T13 shown in (**B**) and DBV-D, K1–11, T14, T15 as well as the outgroups shown in (**C**). The bootstrap analysis of the sequences was 1000 replicates and the cut-off value was 70%. The scale bars show the number of substitutions per base.

All sequences produced in this study were subjected to similarity BLAST searches in the NCBI GenBank databases and nearest matches as well as percent identities can be found in Table 1. The phylogenetic analysis of the 527–528-bp-long partial RT-RNaseH nucleotide sequences showed that the 114 sequences fall within six monophyletic groups according to the suggested classification of yam badnaviruses [3,33,34,39]. The groups include K1, K5, K8, K9, U12 and T13 (Figure 4 and Table 1). A nucleotide percent similarity matrix was generated using MAFFT and is presented in Table S1. We included all DGGE-derived badnavirus sequences in the phylogenetic analysis, as this approach adds another layer of information by giving an approximate indication about the prevalence of a particular sequence in the material tested.

### 3.3. Monophyletic Group Assignment of Sequences Identified in This Study

Five DBV sequences, with each of those derived from individual DGGE bands, clustered into monophyletic group K1 described by Kenyon et al. [3]. Two sequences originating from *D. dumetorum* and *D. esculenta* samples share 99% nucleotide identity to FJ65bDe (AM072660) and FJ75cDe (AM072663), which were sampled from Fiji in 1999 [34]. Three further sequences were isolated from two *D. alata* samples and one *D. dumetorum* sample, showing 99% nucleotide identity to SB42Da (AM072696) isolated from a *D. alata* plant in the Solomon Islands in 2000 [34]. Six DBV sequences clustered into monophyletic group K5, with five of these originating from *D. rotundata* samples and sharing 98–99% nucleotide sequence identity to the eDBV5 clone S1g6Dr (KF829974, [39]) and NGl1950Dr (KX008589, [33]). The latter sequence NGl1950Dr was amplified from a *D. rotundata* plant by rolling circle amplification (RCA) and was considered to therefore most probably be an episomal sequence. Most DGGE-derived sequences (58 out of 114 in total) clustered into monophyletic group K8 and the majority of those originated from *D. rotundata* samples (Figure 4 and Table 1). Twenty-nine out of 114 DBV sequences in total were assigned to monophyletic group K9. Thirteen DBV sequences originating from *D. rotundata* samples clustered into monophyletic group U12 described by Umber et al. [39]. Only one sequence identified by DGGE fell into monophyletic group T13 described in [33]. This sequence (NGb8aDr) appears to be comigrating with DGGE sequence NGb8bDr, which is clustering into monophyletic group K8 and common to most of the *D. rotundata* germplasm tested in this study. A full description of the relationship of sequences is given in Appendix A.

### 3.4. Conservation of Amino Acid Motifs in Partial RT-RNaseH Badnavirus Sequences

BLAST similarity searches of the partial RT-RNaseH coding region derived from DGGE band sequences showed that most of the sequences had close identity to a number of existing badnavirus sequences (Table 1). The analysis of the deduced amino acid sequences of the badnavirus sequences identified in this study, and published sequences of other badnaviruses have shown some distinctive conserved and semi-conserved regions of the family *Caulimoviridae*; the regions represent the ‘FIAVYIDDILVFS’ motif [73,74] at position 17–29 of the deduced protein sequence and the ‘LKTTKGLRSWLGILNYAR’ motif [35] at position 95–112 of the deduced protein sequence of the 528-bp-long partial RT-RNaseH. Moreover, the protein alignment presented in Figure S2 allows the simple identification of single amino acid changes or major differences between the phylogenetic groups. Clear patterns specific to every monophyletic species group can be observed, with for example valine at position 98 present in all sequences clustering into group U12. All protein sequences analysed in this study were compared to the reference sequence of *Dioscorea bacilliform alata virus* (DBALV or DaBVa, X94576-XX94581, [74]).

## 4. Discussion

### 4.1. Potential of DGGE in Badnavirus Diversity Studies and Identification of Potential Integrated Sequences

In this study, the variation in complex (e)DBV sequence mixtures across a subset of the 127 IITA *D. rotundata* samples that scored 100% Badna-positive by PCR in a study by Seal et al. [38] was rapidly and robustly evaluated. The results presented here showed that DGGE is a very useful technique for diversity profiling of the amplified DBV partial RT-RNaseH sequences. This is in agreement with previous reports on the ability of DGGE to differentiate DNA sequences of high similarity in given samples, such as in the diversity studies of viruses, bacteria and phytoplankton [54,75]. A particular strength for the badnavirus sequence diversity is, however, clearly apparent from the similarities in DGGE banding profiles observed between breeding lines of the same *Dioscorea* species indicating highly conserved sequences that appear to represent eDBVs. For example, crossings TDr 89/02475 × TDr 97/00777 and TDr 99/02793 × TDr 1892 show identical DGGE patterns (Figure 3), suggesting the presence of the same set of DBV sequences, with the majority of those likely to be of endogenous nature and ultimately indicating a very similar genetic background of these samples.

The DGGE banding profiles in this study and the resulting clone sequences thereof, depict the vast complexity of DBV sequences present in yam germplasm. The precise analysis of all DBV sequences present in a given sample using DGGE as a method, however, is not straightforward in the light of this complexity and the difficulty of cloning closely migrating bands. Our efforts focused on cloning clearly defined bands. For example, the majority of breeding lines of the same *Dioscorea* species share the common DGGE bands 5, 10 and 11 for *D. rotundata* (Figure 2). Sequences from these bands and others cluster closely together and a total of 22 sequences share 99–100% nucleotide identity to BfA103Dc (AM503393, [34]). It appears probable that these bands may represent integrated sequences as the presence of such high identity episomal viruses in all the different material would not be expected. Episomal badnavirus sequences infecting other crops have been shown to be highly diverse, as illustrated by the genetic diversity of four banana streak virus (BSV) isolates from Australia ranging from 21.8 to 33.6% in a comparison of the amino acid sequences of the ribonuclease H domain in open reading frame 3 (ORF3) [24] and nucleotide diversity of up to 18% of the RT-RNaseH-coding domain of six BSVs from East Africa [41]. Further evidence is that BfA103Dc was shown to be free of episomal viruses by immunosorbent electron microscopy (ISEM) and ELISA [38]. Hybridisation using the common bands as probes in Southern blots would confirm whether DGGE bands 5, 10 and 11 represent common ancient integrated sequence in the host yam genomes rather than an unusually homogeneous episomal viral infection.

The presence of more than one band within every single lane of DGGE analysis reveals that every leaf sample tested contained more than one badnavirus sequence. The presence of sequences representing more than a single badnavirus species in single-leaf samples has been reported before through sequencing of cloned PCR products and generating clones representing several different badnavirus species from a single leaf sample [3,35,60]. This study reveals that in *D. rotundata* this is in fact the norm, rather than an exception as also indicated in our previous study detecting mixed infections using RCA as a tool to amplify episomal badnavirus sequences [33]. In this context we propose DGGE to be used as complementary method to RCA, enabling the rapid identification of potentially integrated sequences while screening for badnavirus diversity.

Testing of DGGE as a tool in the analysis of complex badnavirus sequence mixtures present in samples of yam breeding lines in this study was based on the assumption that the previous procedure by cloning and sequencing of PCR products is an inefficient method to reveal full sequence diversity. Such techniques require the selection and sequencing of a large number of clones in order to unravel all sequence diversity present in a given sample as demonstrated also for BSV [76]. Equally, RFLP was reported not much a useful tool for yam badnavirus diversity, since sequences with different RFLP patterns were found clustered in the same species group [35]. The above illustrates that the practical advantage of DGGE is that it is a rapid means of detecting sequence diversity in uniformly sized PCR products, eliminating the need for labour-intensive screening of redundant clones [77]. Equally, in a situation when there are a large number of samples to be analysed that contain multiple badnavirus sequences, DGGE is the most appropriate technique in terms of cost and practicality for a diversity study compared to direct sequencing of clones following PCR or RFLP.

Conversely, DGGE suffers from some methodical pitfalls and inherent practical limitations. One concern is the detection limit, with Muyzer et al. [45] suggesting that any target DNA that is less than 1% of the total target pool is unlikely to be detected by DGGE. As such, low badnavirus titers could lead to the unlikely detection of true episomal DBV sequences by DGGE, compared to endogenous sequences potentially existing in higher copy numbers in some yam germplasm. As a related caveat, discrete fingerprint bands may not always be apparent when analysing highly diverse samples, leading to smearing or poorly resolved patterns [44]. Another related limitation is the comigration of DNA molecules of different sequence as reported by Ercolini [49] and also experienced in this study. Although DGGE was able to separate sequences with high similarities, problems included accurate cutting of closely-migrating DGGE bands in particular under suboptimal resolution due to the occurrence of smeared backgrounds as can be seen in several samples presented in Figure 3. Additionally, some technical problems were encountered in carrying out the DGGE technique, namely general handling of fragile DGGE gels and the persistent trouble of background smearing in gels that made band-scoring problematic. Several modifications were performed on the PCR conditions, ranging from altering concentrations of primers, the choice of the *Taq* polymerase enzyme, the DGGE gradient range and the volume of PCR products loaded per gel lane. In this context, we found the procedural comments on the DGGE technique given by Green et al. [44] useful. Different primers (Badna-FP versus Badna-RP GC-clamped primer) were also tried, but the comparison shown in Figure 2 concluded a limited suitability of the Badna-RP GC-clamp primer in the DBV diversity study because of the potential under-representation of badnavirus sequences clustering in the K9 monophyletic group.

For the future, a worthwhile additional improvement would be the inclusion of a reference DGGE standard in which a profile is created using pooled clones from distinct DGGE bands. Here, clone sequences or PCR re-amplification products would be analysed by DGGE in a lane adjacent to the original sample PCR product from which it was excised. This will assist the typing of unknown populations and eliminate the need for DGGE band cloning and subsequent sequencing. Moreover, the degeneracy of the Badna-FP/-RP primers is expected to contribute to the poor resolution (smearing experienced in this study), potentially creating multiple products per template sequence differing only in their primer binding site. Hence, the design of monophyletic group specific primers should be considered in the future, potentially improving DGGE resolution while reducing the complexity at the same time by focusing the analysis on the most prevalent and interesting DBV species.

### 4.2. DGGE-Captured Badnavirus Diversity

To investigate the in-depth diversity of badnavirus sequences present in the yam samples analysed in this study, DGGE analysis was applied and representative bands were cloned and sequenced. All sequenced bands were shown to be partial RT-RNaseH sequences of badnaviruses, with 112 DBV sequences analysed clustering into six (K1, K5, K8, K9, U12 and T13) of the 15 putative species groups identified by Bömer et al. [33], Bousalem et al. [34], Kenyon et al. [3] and Umber et al. [39]. A comparison of their amino acid sequences generated from the translated partial RT-RNaseH region sequences together with other members of the family *Caulimoviridae* revealed conserved and semi-conserved regions similar to the previously published badnavirus sequences [3,33,34,35,38,73,78,79,80]. In particular, a stretch of the conserved ‘FIAVYIDDILVFS’ region of the RT in the C-terminal of the ORF3 polyprotein was observed, as was the semi-conserved ‘LKTTKGLRSWLGILNYAR’ region (Figure S2). This confirms that DNA extracted from *Dioscorea* spp. samples contained sequences belonging to the genus *Badnavirus* of the family *Caulimoviridae* [73,78,79,80,81].

Sequence analyses of the partial RT-RNaseH domain in this work support the classification and diversity of yam badnaviruses proposed by Kenyon et al. [3], and subsequently substantiated upon by Bousalem et al. [34], Umber et al. [39] and Bömer et al. [33]. The maximum variability recorded within the partial RT-RNaseH coding region at the nucleotide level among the 112 yam badnavirus sequences determined in this study was 37%. This degree of variability was within the range of intergroup nucleotide diversity for group K1–K11 (23.1–39.4%) of yam badnaviruses [3]. For badnaviruses present in other plant hosts, high diversity levels have also been reported, with 21.8–33.6% sequence diversity in a comparison of the amino acid sequences of the ribonuclease H domain in ORF3 reported for four BSV isolates from Australia [24], 28% maximum nucleotide diversity for Ugandan BSV isolates [2], 29.4% maximum nucleotide diversity between complete genomes of CSSV isolates [82], and up to 33.5% nucleotide variability within the partial RT-RNaseH sequences for isolates of sugarcane bacilliform viruses (SCBVs) [5].

Two sequences (NGl65De and NGb53Dr) determined in this study shared <60% nucleotide identity with any previously identified yam badnavirus sequence group but were similar to the divergent sequences groups of K12 and K13 reported by Kenyon et al. [3] from South Pacific yams. The sequences clustered closer to RTBV, a more distantly related virus in the family *Caulimoviridae*, than any member of the genus *Badnavirus*. It is possible that these sequence groups represent divergent badnaviruses, ancient endogenous pararetrovirus sequences or new genera within the family *Caulimoviridae* [3]. Further research is needed to characterise the nature of these sequences.

### 4.3. Endogenous Badnavirus Partial RT-RNaseH Sequences

To date, four of the 15 yam badnavirus species groups identified have been reported to contain eDBV sequences, namely K5, K8, K9 and U12 [38,39]. Phylogenetic analyses of the cloned DGGE bands revealed that all the sequences that originated from DGGE bands common to the majority of *D. rotundata* (for example NGb30bDr and NGb1aDr) samples clustered into either the K8 or the K9 species groups (Figure 4). These common bands show 99–100% nucleotide identity to several more sequences identified in this study as well as integrated DBV sequences described by Umber et al. [39], and hence, it seems probable that they represent eDBV sequences. A number of DGGE band sequences from *D. rotundata* were found to both share 99% nucleotide identity to eDBV9 clone S1h2Dr (KF829975, [39]) or eDBV8 clone S2h9Dr (KF829997, [39]) respectively, suggesting that these sequences represent integrated sequences in the samples tested. Several more DGGE band sequences present in *D. rotundata* were found that clustered with the other two yam badnavirus/eDBV species groups (K5 and U12) (Figure 4). For example, sequences NGl9a/bDr, NGl12a/bDr, and NGl13a/bDr of the U12 species group were from bands that were present in several *D. rotundata.* They share 99–100% nucleotide identity to eDBV12 clone S1a4Dr (KF829956, [39]), suggesting these sequences could also be integrated sequences in the samples tested. Equally, NGb22a/bDr, NGb23aDr, NGb24aDr, NGb52Dr could also represent integrated sequences as the sequences share 98–99% nucleotide identity to eDBV5 clone S1g6Dr (KF829974, [39]).

In summary, 28 sequences showed eDBV sequences as their nearest match with nucleotide identities above 99%. Twenty-two sequences were also identified that have 99–100% identity to the badnavirus-particle free BfA103Dc (AM503393, [34]) and as a result are likely to represent integrated sequences as described above. Additionally, a further 32 sequences clustering into monophyletic group K8 are highly conserved among themselves (GyJT2DT, AM503389, [34], being the nearest match), suggesting that these sequences are also integrated. These findings together lead to at least 82 of 112 (72%) sequences in this study considered to most probably originate from integrated sequences. It is proposed that DGGE therefore is very effective at revealing eDBV sequences when testing plant germplasm.

In this study, the breeding lines were derived from true seed yam, and this material has been grown over successive cropping seasons at the IITA. The true seed material should have been free of episomal badnavirus particles, as yam badnaviruses have not been reported to be seed-transmissible [62]. However, the origin from true seed yam does not automatically exclude that episomal forms of virus can be present. Vertical transmission of integrated copies via seeds is possible as reported for endogenous PVCV in petunia, where the integrated virus gets activated by direct transcription of integrated PVCV sequences in the form of a tandem array [22]. Vertical transmission of activatable eDBVs cannot be excluded in yams and more research on this potential risk is needed. Nevertheless, the presence of all the common DGGE bands in yam lines analysed in this study suggests that these most likely represent eDBV sequences, whereas additional DGGE bands detected only in some lines potentially represent new infections during the propagation of the material in the field over many cultivation seasons. Further analysis of the DGGE sequences using the Southern blot technique will help to determine if these sequences are diverse eDBVs or represent episomal viruses acquired through screen-house and field propagation.

## 5. Conclusions

In summary, a workflow combining PCR and DGGE methods for rapid and efficient determination and unravelling of complex mixtures of potentially episomal and endogenous badnavirus sequences has been developed (Figure 1). Here, we used specific PCR primers to amplify a partial sequence of the badnavirus RT-RNaseH coding region to investigate the molecular diversity of (e)DBV sequences in selected West African yam germplasm. PCR products were resolved using DGGE, giving characteristic banding patterns for each yam line examined. This approach is described here for the first time for the assessment of badnavirus sequences present in *Dioscorea* spp. germplasm and resulted in the identification of complex DGGE profiles representing multiple sequences of DBVs. A total of 112 yam badnavirus sequences were generated (GenBank accession numbers KY555456 to KY555569), falling into six monophyletic species groups. The conserved nature of several DGGE-derived yam badnavirus sequences, as well as high nucleotide identities to eDBV sequences deposited in GenBank suggests that the majority of West African yam germplasm contains a mixture of integrated badnavirus sequences. Our findings highlight DGGE as an extremely useful technique for rapid indication of badnavirus sequence diversity in such samples containing multiple eDBVs, enabling a snapshot of the diversity between genomes to be taken. The approach taken in this study to enable rapid identification of potential candidate integrated badnavirus sequences, indicated by their conserved nature across germplasm, should have wide application for the study of an ever-increasing number of plant species found to contain this previously understudied, but important genus of plant viruses.

## Figures and Tables

**Table 1:** Basic local alignment search tool (BLAST) analysis of partial RT-RNaseH sequences cloned from DGGE bands.

Plant Accession ^a^	DGGE Sequence ^b^	Accession	Primers ^c^	Size (bp)	NCBI Nearest Match	Identity (%)	Species Group ^d^
TDr 89/02475	NGb1aDr	KY555456	BF-GC + BR	528	eDBV9_S1h6Dr (KF829977)	99	K9
TDr 89/02475	NGb1bDr	KY555457	BF-GC + BR	528	432B39Ds (AM503361)	99	K9
TDr 89/02475	NGb2aDr	KY555458	BF-GC + BR	528	432B39Ds (AM503361)	100	K9
TDr 89/02475	NGb2bDr	KY555459	BF-GC + BR	528	432B39Ds (AM503361)	99	K9
TDr 89/02475	NGb3aDr	KY555460	BF-GC + BR	527	432B39Ds (AM503361)	99	K9
TDr 89/02475	NGb3bDr	KY555461	BF-GC + BR	528	432B39Ds (AM503361)	99	K9
TDr 89/02475	NGb4Dr	KY555462	BF-GC + BR	528	GyJT2Dt (AM503389)	93	K8
TDr 89/02475	NGb5Dr	KY555463	BF-GC + BR	528	BfA103Dc (AM503393)	99	K8
TDr 89/02475	NGb6aDr	KY555464	BF-GC + BR	528	GyJT2Dt (AM503389)	92	K8
TDr 89/02475	NGb6bDr	KY555465	BF-GC + BR	528	GyJT2Dt (AM503389)	93	K8
TDr 89/02475	NGb7aDr	KY555466	BF-GC + BR	528	GyJT2Dt (AM503389)	92	K8
TDr 89/02475	NGb7bDr	KY555467	BF-GC + BR	528	GyJT2Dt (AM503389)	92	K8
TDr 89/02475	NGb8aDr	KY555468	BF-GC + BR	528	DBRTV1-[3RT] (KX008598)	99	T13
TDr 89/02475	NGb8bDr	KY555469	BF-GC + BR	528	GyJT2Dt (AM503389)	92	K8
Pona	NGl9aDr	KY555470	BF-GC + BR	528	eDBV12_S1a4Dr (KF829956)	99	U12
Pona	NGl9bDr	KY555471	BF-GC + BR	528	eDBV12_S1a4Dr (KF829956)	99	U12
Pona	NGl10aDr	KY555472	BF + BR-GC	528	BfA103Dc (AM503393)	99	K8
Pona	NGl10bDr	KY555473	BF + BR-GC	528	BfA103Dc (AM503393)	99	K8
Pona	NGl11aDr	KY555474	BF + BR-GC	528	BfA103Dc (AM503393)	99	K8
Pona	NGl11bDr	KY555475	BF + BR-GC	528	BfA103Dc (AM503393)	99	K8
Pona	NGl12aDr	KY555476	BF + BR-GC	528	eDBV12_S1a4Dr (KF829956)	99	U12
Pona	NGl12bDr	KY555477	BF + BR-GC	528	eDBV12_S1a4Dr (KF829956)	100	U12
Pona	NGl13aDr	KY555478	BF + BR-GC	528	eDBV12_S1a4Dr (KF829956)	100	U12
Pona	NGl13bDr	KY555479	BF + BR-GC	528	eDBV12_S1a4Dr (KF829956)	99	U12
Pona	NGl14aDr	KY555480	BF + BR-GC	528	BfA103Dc (AM503393)	99	K8
Pona	NGl14bDr	KY555481	BF + BR-GC	528	BfA103Dc (AM503393)	100	K8
Pona	NGl15aDr	KY555482	BF + BR-GC	528	BfA103Dc (AM503393)	99	K8
Pona	NGl15bDr	KY555483	BF + BR-GC	528	BfA103Dc (AM503393)	99	K8
TDr 89/02475	NGb16aDr	KY555484	BF + BR-GC	528	GyJT2Dt (AM503389)	92	K8
TDr 89/02475	NGb16bDr	KY555485	BF + BR-GC	527	432B39Ds (AM503361)	99	K9
TDr 89/02475	NGb17aDr	KY555486	BF + BR-GC	528	GyJT2Dt (AM503389)	92	K8
TDr 89/02475	NGb17bDr	KY555487	BF + BR-GC	528	GyJT2Dt (AM503389)	92	K8
TDr 89/02475	NGb18aDr	KY555488	BF + BR-GC	528	BfA103Dc (AM503393)	99	K8
TDr 89/02475	NGb18bDr	KY555489	BF + BR-GC	528	GyJT2Dt (AM503389)	93	K8
TDr 89/02475	NGb19aDr	KY555490	BF + BR-GC	528	BfA103Dc (AM503393)	99	K8
TDr 89/02475	NGb19bDr	KY555491	BF + BR-GC	528	BfA103Dc (AM503393)	99	K8
TDr 89/02475	NGb20aDr	KY555492	BF + BR-GC	528	GyJT2Dt (AM503389)	92	K8
TDr 89/02475	NGb20bDr	KY555493	BF + BR-GC	528	GyJT2Dt (AM503389)	93	K8
TDr 95/18544	NGb21aDr	KY555494	BF + BR-GC	528	BfA103Dc (AM503393)	99	K8
TDr 95/18544	NGb21bDr	KY555495	BF + BR-GC	528	BfA103Dc (AM503393)	99	K8
TDr 95/18544 (o.p.)	NGb22aDr	KY555496	BF-GC + BR	528	eDBV5_S1g6Dr (KF829974)	99	K5
TDr 95/18544 (o.p.)	NGb22bDr	KY555497	BF-GC + BR	528	eDBV5_S1g6Dr (KF829974)	99	K5
TDr 95/18544 (o.p.)	NGb23aDr	KY555498	BF-GC + BR	528	eDBV5_S1g6Dr (KF829974)	99	K5
TDr 95/18544 (o.p.)	NGb23bDr	KY555499	BF-GC + BR	528	BfA103Dc (AM503393)	97	K8
TDr 95/18544 (o.p.)	NGb24aDr	KY555500	BF-GC + BR	528	eDBV5_S1g6Dr (KF829974)	99	K5
TDr 95/18544 (o.p.)	NGb24bDr	KY555501	BF-GC + BR	528	BfA103Dc (AM503393)	99	K8
TDr 97/00917 × TDr 99/02607	NGb25aDr	KY555502	BF-GC + BR	528	eDBV9_S1h2Dr (KF829975)	99	K9
TDr 97/00917 × TDr 99/02607	NGb25bDr	KY555503	BF-GC + BR	528	BN4Dr (AM944586)	99	K9
TDr 97/00917 × TDr 99/02607	NGb26aDr	KY555504	BF-GC + BR	528	BN4Dr (AM944586)	99	K9
TDr 97/00917 × TDr 99/02607	NGb26bDr	KY555505	BF-GC + BR	528	eDBV9_G1Dr (KF830002)	99	K9
TDr 97/00917 × TDr 99/02607	NGb27aDr	KY555506	BF-GC + BR	528	eDBV12_S2a7Dr (KF829978)	99	U12
TDr 97/00917 × TDr 99/02607	NGb27bDr	KY555507	BF-GC + BR	528	BN4Dr (AM944586)	99	K9
TDr 97/00917 × TDr 99/02607	NGb28aDr	KY555508	BF-GC + BR	528	BN4Dr (AM944586)	99	K9
TDr 97/00917 × TDr 99/02607	NGb28bDr	KY555509	BF-GC + BR	528	BN4Dr (AM944586)	99	K9
TDr 97/00917 × TDr 99/02607	NGb29aDr	KY555510	BF-GC + BR	528	BN4Dr (AM944586)	99	K9
TDr 97/00917 × TDr 99/02607	NGb29bDr	KY555511	BF-GC + BR	528	eDBV9_S2f8Dr (KF829993)	99	K9
TDr 97/00917 × TDr 99/02607	NGb30aDr	KY555512	BF-GC + BR	527	BN4Dr (AM944586)	99	K9
TDr 97/00917 × TDr 99/02607	NGb30bDr	KY555513	BF-GC + BR	528	BfA103Dc (AM503393)	100	K8
TDr 99/02793 × TDr 1892	NGb31Dr	KY555514	BF-GC + BR	528	GyJT2Dt (AM503389)	93	K8
TDr 99/02793 × TDr 1892	NGb32Dr	KY555515	BF-GC + BR	528	eDBV9_S1e3Dr (KF829969)	100	K9
TDr 04/219 × TDr 98/02677	NGb33aDr	KY555516	BF-GC + BR	527	GyJT2Dt (AM503389)	92	K8
TDr 04/219 × TDr 98/02677	NGb33bDr	KY555517	BF-GC + BR	528	GyJT2Dt (AM503389)	93	K8
TDr 97/00917 (o.p.)	NGb34aDr	KY555518	BF-GC + BR	528	GyJT2Dt (AM503389)	92	K8
TDr 97/00917 (o.p.)	NGb34bDr	KY555519	BF-GC + BR	528	GyJT2Dt (AM503389)	93	K8
TDr 96/00629 × TDr 99/02607	NGb35aDr	KY555520	BF-GC + BR	528	432B39Ds (AM503361)	99	K9
TDr 96/00629 × TDr 99/02607	NGb35bDr	KY555521	BF-GC + BR	528	432B39Ds (AM503361)	100	K9
TDr 97/00917 × TDr 99/02607	NGb36aDr	KY555522	BF-GC + BR	528	BN4Dr (AM944586)	99	K9
TDr 97/00917 × TDr 99/02607	NGb36bDr	KY555523	BF-GC + BR	527	BN4Dr (AM944586)	99	K9
TDr 97/00917 × TDr 99/02607	NGb37aDr	KY555524	BF-GC + BR	528	eDBV9_S2f8Dr (KF829993)	99	K9
TDr 97/00917 × TDr 99/02607	NGb37bDr	KY555525	BF-GC + BR	528	eDBV9_S1h2Dr (KF829975)	99	K9
TDr 04/219 × TDr 04/219	NGb38aDr	KY555526	BF-GC + BR	528	GyJT2Dt (AM503389)	93	K8
TDr 04/219 × TDr 04/219	NGb38bDr	KY555527	BF-GC + BR	528	GyJT2Dt (AM503389)	92	K8
TDr 04/219 × TDr 04/219	NGb39aDr	KY555528	BF-GC + BR	528	GyJT2Dt (AM503389)	92	K8
TDr 04/219 × TDr 04/219	NGb39bDr	KY555529	BF-GC + BR	528	GyJT2Dt (AM503389)	93	K8
TDr 04/219 × TDr 04/219	NGb40Dr	KY555530	BF-GC + BR	528	GyJT2Dt (AM503389)	93	K8
TDr 97/00205 ×TDr 1892	NGb41aDr	KY555531	BF-GC + BR	528	eDBV12_S1a4Dr (KF829956)	99	U12
TDr 97/00205 ×TDr 1892	NGb41bDr	KY555532	BF-GC + BR	528	eDBV9_S1h2Dr (KF829975)	99	K9
TDr 96/00629 × TDr 99/02607	NGb42aDr	KY555533	BF-GC + BR	528	eDBV12_S1a4Dr (KF829956)	99	U12
TDr 96/00629 × TDr 99/02607	NGb42bDr	KY555534	BF-GC + BR	528	432B39Ds (AM503361)	99	K9
TDr 95/18544	NGb43Dr	KY555535	BF-GC + BR	528	BfA103Dc (AM503393)	99	K8
TDr 95/18544	NGb44Dr	KY555536	BF-GC + BR	528	BfA103Dc (AM503393)	99	K8
TDr 95/18544	NGb45Dr	KY555537	BF-GC + BR	528	BfA103Dc (AM503393)	99	K8
TDr 96/00629	NGb46aDr	KY555538	BF-GC + BR	528	eDBV12_S2a7Dr (KF829978)	99	U12
TDr 96/00629	NGb46bDr	KY555539	BF-GC + BR	528	BfA103Dc (AM503393)	99	K8
TDr 89/02677	NGb47aDr	KY555540	BF-GC + BR	528	GyJT2Dt (AM503389)	92	K8
TDr 89/02677	NGb47bDr	KY555541	BF-GC + BR	528	GyJT2Dt (AM503389)	93	K8
TDr 89/02677	NGb48aDr	KY555542	BF-GC + BR	528	GyJT2Dt (AM503389)	92	K8
TDr 89/02677	NGb48bDr	KY555543	BF-GC + BR	528	GyJT2Dt (AM503389)	92	K8
TDr 89/02677	NGb49Dr	KY555544	BF-GC + BR	528	GyJT2Dt (AM503389)	92	K8
TDr 96/00629	NGb50Dr	KY555545	BF-GC + BR	528	eDBV12_S2a7Dr (KF829978)	99	U12
TDr 89/02677	NGb51Dr	KY555546	BF-GC + BR	528	GyJT2Dt (AM503389)	91	K8
TDr 97/00917 (o.p.)	NGb52Dr	KY555547	BF-GC + BR	528	eDBV5_S1g6Dr (KF829974)	98	K5
TDr 97/00917 (o.p.)	NGb53Dr	KY555548	BF-GC + BR	528	NC1 (KJ854414)	79	K13
TDr 97/00917 × TDr 99/02607	NGb54Dr	KY555549	BF-GC + BR	528	eDBV9_S1h2Dr (KF829975)	99	K9
TDr 99/02793 (o.p.)	NGb55aDr	KY555550	BF-GC + BR	528	BfA103Dc (AM503393)	100	K8
TDr 99/02793 (o.p.)	NGb55bDr	KY555551	BF-GC + BR	528	BfA103Dc (AM503393)	99	K8
TDr 96/00621 (o.p.)	NGb56Dr	KY555552	BF-GC + BR	528	eDBV12_S1a4Dr (KF829956)	100	U12
TDr 97/00917 × TDr 97/00777	NGb57Dr	KY555553	BF-GC + BR	528	GyJT2Dt (AM503389)	93	K8
TDr 96/00629 × TDr 99/02607	NGb58Dr	KY555554	BF-GC + BR	528	GyJT2Dt (AM503389)	92	K8
TDr 97/00917 × TDr 99/02607	NGb59Dr	KY555555	BF-GC + BR	528	eDBV12_S2h10Dr (KF829998)	99	U12
TDa 99/00240 × TDa 95/00310	NGb60Da	KY555556	BF-GC + BR	528	GyJT2Dt (AM503389)	92	K8
TDa 99/00240 × TDa 01/00012	NGb61Da	KY555557	BF-GC + BR	528	NG1Da (AM944571)	93	K8
TDa 01/00081 × TDa 02/00012	NGb62Da	KY555558	BF-GC + BR	528	GyJT2Dt (AM503389)	93	K8
TDa 1/00081×TDa 98/00150	NGb63Da	KY555559	BF-GC + BR	528	SB42Da (AM072696)	99	K1
TDa 00/00194 × TDa 98/00150	NGb64Da	KY555560	BF-GC + BR	528	eDBV9_S1b6Dr (KF829960)	89	K9
TDe 3049A	NGl65De	KY555561	BF-GC + BR	528	WS31aDn (AM421696)	73	K12
TDd 4118B	NGl66Dd	KY555562	BF-GC + BR	528	eDBV5_S1un5Dr (KF830000)	93	K5
TDd 4118B	NGl67Dd	KY555563	BF-GC + BR	528	BN2Da (AM944584)	98	K8
TDr 1950B	NGl68Dr	KY555564	BF-GC + BR	528	GN2Dr (AM944575)	96	K8
TDc 3808C	NGl69Dc	KY555565	BF-GC + BR	528	eDBV9_S1b2Dr (KF829958)	100	K9
TDb 3045B	NGl70Db	KY555566	BF-GC + BR	528	SB42Da (AM072696)	99	K1
TDd 3778B	NGl71Dd	KY555567	BF-GC + BR	528	FJ65bDe (AM072660)	99	K1
TDa 1013C	NGl72Da	KY555568	BF-GC + BR	528	SB42Da (AM072696)	99	K1
TDe 3028A	NGl73De	KY555569	BF-GC + BR	528	FJ65bDe (AM072660)	99	K1

^a^ The host plants are represented by plant accession. TDa: *Dioscorea alata* accession; TDb: *Dioscorea bulbifera* accession; TDc: *Dioscorea cayenensis* accession; TDd: *Dioscorea dumetorum* accession; TDe: *Dioscorea esculenta* accession; TDr: *D. rotundata* accession; o.p.: open pollinated; ^b^ The DGGE clone sequences were coded as follows: the first two letters stand for the country of origin (NG = Nigeria), ‘b’ represents breeding line samples, ‘l’ represents landrace yam samples, the middle number denotes the position of the excised DGGE band, the next letter denotes the clone (a = clone a and b = clone b) and the last two letters refer to the *Dioscorea* host species (e.g., Dr = *Dioscorea rotundata*); ^c^ BF: Badna FP; BR = Badna-RP; GC: GC clamp (see Methods for details); eDBV: endogenous *Dioscorea* bacilliform viruses. ^d^ According to phylogenetic tree (Figure 4).

## Supplementary Materials

The following are available online at www.mdpi.com/1999-4915/9/7/181/s1, Figure S1: Denaturing gradient gel electrophoresis (DGGE) analysis of partial RT-RNaseH badnavirus sequences from eleven samples consisting of cross-breeding lines of *D. alata*, Figure S2: Protein alignment from deduced amino acid sequences of partial RT-RNaseH nucleotide sequences of 114 yam badnavirus sequences determined in this study together with other members of the family *Caulimoviridae*, Table S1: Nucleotide percent similarity matrix.

## Abbreviations

The following abbreviations are used in this manuscript:

BF	Badna-forward primer
BLAST	basic local alignment search tool
BR	Badna-reverse primer
BSOLV	*Banana streak OL virus*
BSV	banana streak virus
ComYMV	*Commelina yellow mottle virus*
CSSV	*Cacao swollen shoot virus*
CTAB	cetyltrimethylammonium bromide
DBALV	*Dioscorea bacilliform alata virus*
DBRTV	*Dioscorea bacilliform rotundata* (RT) *virus*
DBV	Dioscorea bacilliform virus
DGGE	denaturing gradient gel electrophoresis
DOAJ	Directory of open access journals
eDBV	endogenous Dioscorea bacilliform virus
GC	GC clamp
ICTV	International Committee on Taxonomy of Viruses
IITA	International Institute of Tropical Agriculture
ISEM	immunosorbent electron microscopy
LD	linear dichroism
MAFFT	Multiple Alignment using Fast Fourier Transform
NCBI	National Centre for Biotechnology Information
NG	Nigeria
NGS	next-generation sequencing
NRI	Natural Resources Institute
o.p.	open pollinated
ORF	open reading frame
PCR	polymerase chain reaction
RCA	rolling circle amplification
RFLP	restriction fragment length polymorphism
RNaseH	ribonuclease H
RT	reverse transcriptase
RTBV	*Rice tungro bacilliform virus*
SCBMOV	*Sugarcane bacilliform MO virus*
TaBV	*Taro bacilliform virus*
TAE	Tris-acetate-EDTA
TBE	Tris-Boric acid-EDTA
TDa	*Dioscorea alata* accession
TDb	*Dioscorea bulbifera* accession
TDc	*Dioscorea cayenensis* accession
TDe	*Dioscorea esculenta* accession
TDr	*Dioscorea rotundata* accession

## Appendix A

Five DBV sequences, with each of those derived from individual DGGE bands, clustered into monophyletic group K1 described by Kenyon et al. [3]. Sequences NGl71Dd isolated from *D. dumetorum* and NGl73De from *D. esculenta* both share 99% nucleotide identity to FJ65bDe (AM072660) and FJ75cDe (AM072663), which were sampled from Fiji in 1999 by Lebas and described by Bousalem et al. [34] as representative sequences of the DeBV-A sub-group A (Dioscorea esculenta bacilliform virus A) monophyletic group. Sequences NGb63Da and NGl72Da isolated from *D. alata*, as well as NGl70Db from *D. dumetorum* show 99% nucleotide identity to SB42Da (AM072696) isolated from a *D. alata* plant in the Solomon Islands in 2000 by Lebas and described by Bousalem et al. [34] as a representative sequence of the DeBV-A sub-group B.

Six DBV sequences clustered into monophyletic group K5, with five of these originating from *D. rotundata* samples (NGb52Dr, NGb22a/bDr, NGb23aDr and NGb24aDr) and sharing 98–99% nucleotide sequence identity to eDBV5 clone S1g6Dr (KF829974, [39]) and NGl1950Dr (KX008589, [33]). The latter sequence NGl1950Dr was amplified from a *D. rotundata* plant by rolling circle amplification (RCA) in our previous study [33] and was considered to therefore most probably be an episomal sequence. Another DGGE-derived sequence (NGl66Dd) isolated from *D. dumetorum* shows 93% nucleotide sequence identity to eDBV5 clone S1un5Dr (KF830000, [39]).

Most DGGE-derived sequences (58 out of 114 in total) clustered into monophyletic group K8 and the majority of those originated from *D. rotundata* samples (Figure 4 and Table 1). Twenty-three DBV sequences assigned to K8 and isolated from *D. rotundata* samples cluster very closely together and share 97–100% nucleotide identity to BfA103Dc (AM503393, [34]) and NGb0477Dr (KX008586, [33]). Sequence NGb0477Dr was amplified by RCA in our previous study showing 99% nucleotide identity to BfA103Dc, Gn1633Dr, Gn845Dr and Gn502Dr [33]. Sequence BfA103Dc (AM503393, [34]) was derived from a Pilimpikou yam sample which was reported as viral particle-free, whereas the other three sequences derived from yam samples of Guinea in which badnavirus particles were detected using ISEM in a previous study by Seal et al. [38]. Another K8 sequence (NGl67Dd) originating from *D. dumetorum* is 98% identical to BN2Da from Benin (AM944584, [35]), while sequence NGl68Dr shares 96% identity with GN2Dr from Ghana (AM944575, [35]), and sequence NGb61Da is 93% identical to NG1Da from Nigeria (AM944571, [35]). Moreover, 32 DGGE-derived sequences share 91–93% nucleotide identity to GyJT2Dt isolated in French Guiana in 1998 (AM503389, [34]).

Twenty-nine out of 114 DBV sequences in total were assigned to monophyletic group K9. Of those, nine DGGE-derived sequences originating from *D. rotundata* samples share 99% nucleotide sequence identity to BN4Dr (AM944586, [35]) isolated from a *D. rotundata* plant in Benin. A further four sequences (NGb25aDr, NGb37bDr, NGb54Dr and NGb41bDr) isolated from *D. rotundata* are 99% identical to eDBV9 clone S1h2Dr (KF829975, [39]), and two more sequences (NGb29bDr and NGb37aDr) from *D. rotundata* samples are 99% identical to eDBV9 clone S2f8Dr (KF829993, [39]). Moreover, nine DGGE-derived sequences (NGb1bDr, NGb2a/bDr, NGb3a/bDr, NGb35a/bDr, NGb42bDr and NGb16bDr) isolated from *D. rotundata* samples share 99–100% identity with 432B39Ds (AM503361, [34]) derived from *D. sansibarensis* isolated in Benin 1999 in which badnavirus particles were detected using ISEM [38] and FJ60aDr (AM072658, [34]) from *D. rotundata* isolated in Fiji 1999 by Lebas. A further five sequences are 89–100% identical to either eDBV9 clone G1Dr (KF830002, [39]), eDBV9 clone S1h6Dr (KF829977, [39]), eDBV9 clone S1b6Dr (KF829960, [39]), eDBV9 clone S1e3Dr (KF829969, [39]) or eDBV9 clone S1b2Dr (KF829958, [39]), respectively.

Thirteen DBV sequences originating from *D. rotundata* samples clustered into monophyletic group U12 as described by Umber et al. [39]. Nine of these sequences (NGl9a/bDr, NGl12a/bDr, NGl13a/bDr, NGb41aDr, NGb42aDr and NGb56Dr) are 99–100% identical to eDBV12 clone S1a4Dr (KF829956, [39]) and GHL24Dr isolated in Ghana 1995 (AM072665, [34]). Three further sequences are 99% identical to eDBV12 clone S2a7Dr (KF829978, [39]) and Mt818Dr isolated in Martinique 1998 (AM503378, [34]). The remaining sequence NGb59Dr is 99% identical to eDBV12 clone S2h10Dr (KF829998, [39]).

Only one sequence identified by DGGE fell into the monophyletic group T13 described in our previous study [33]. Sequence NGb8aDr has 99% identity to the episomal Dioscorea bacilliform rotundata (RT) virus 1 (DBRTV1) sequence originating from *D. rotundata* (KX008596, [33]), which is also available as a full genome sequence (GenBank KX008574). However, the second clone of this DGGE band clustered into group K8, indicating cross-contamination with a closely migrating band.
