# Supplementary material for: PCR-DGGE Analysis: Unravelling Complex Mixtures of Badnavirus Sequences Present in Yam Germplasm
Source: Viruses. 2017 Jul 11;9(7):181. doi: 10.3390/v9070181 (PMC5537673; doi:10.3390/v9070181)
Supplement: Supplementary file 1 [file viruses-09-00181-s001.zip › Figure S1.docx]

**
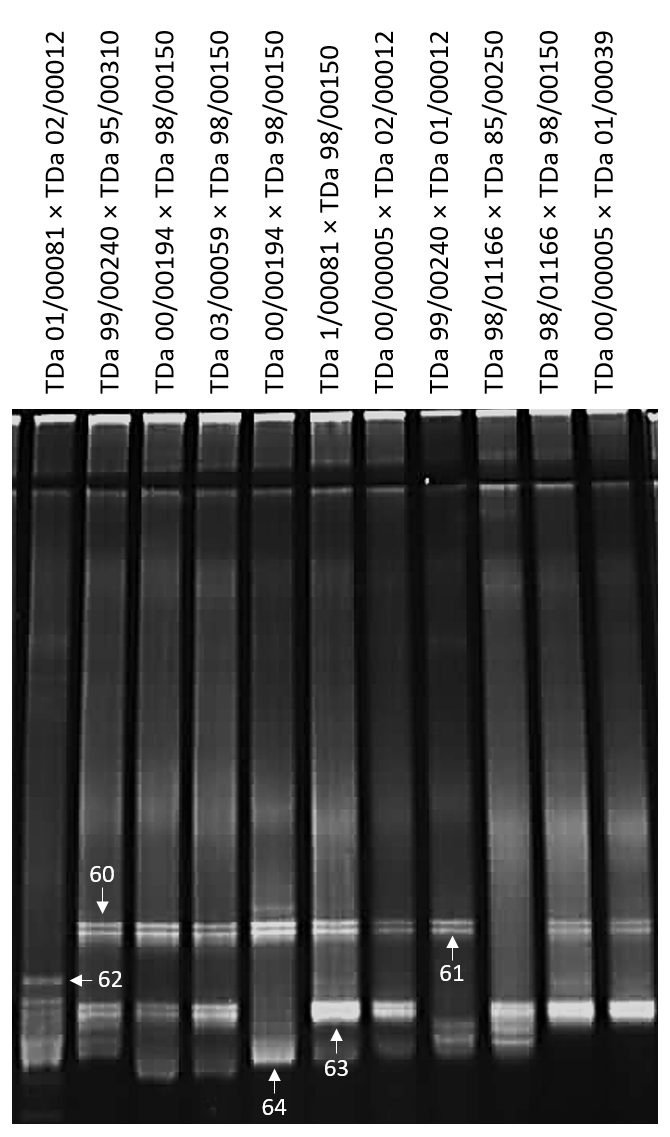
**

**Figure S1.** Denaturing gradient gel electrophoresis (DGGE) analysis of partial RT-RNaseH badnavirus sequences from eleven samples consisting of cross-breeding lines of *D. alata* comparing patterns of PCR amplifications (20 µl loaded) using the generic badnavirus primer pair Badna-FP/-RP with a GC-clamp fused to the forward primer. The denaturing gradient was 30-55% and DGGE was performed at 80 V at a temperature of 60 ˚C for 18 h. Band numbers 60–64 were excised and cloned. The corresponding sequences are presented in Table 1.
