# Supplementary material for: PCR-DGGE Analysis: Unravelling Complex Mixtures of Badnavirus Sequences Present in Yam Germplasm
Source: Viruses. 2017 Jul 11;9(7):181. doi: 10.3390/v9070181 (PMC5537673; doi:10.3390/v9070181)
Supplement: Supplementary file 1 [file viruses-09-00181-s001.zip › Figure S2.docx]

**
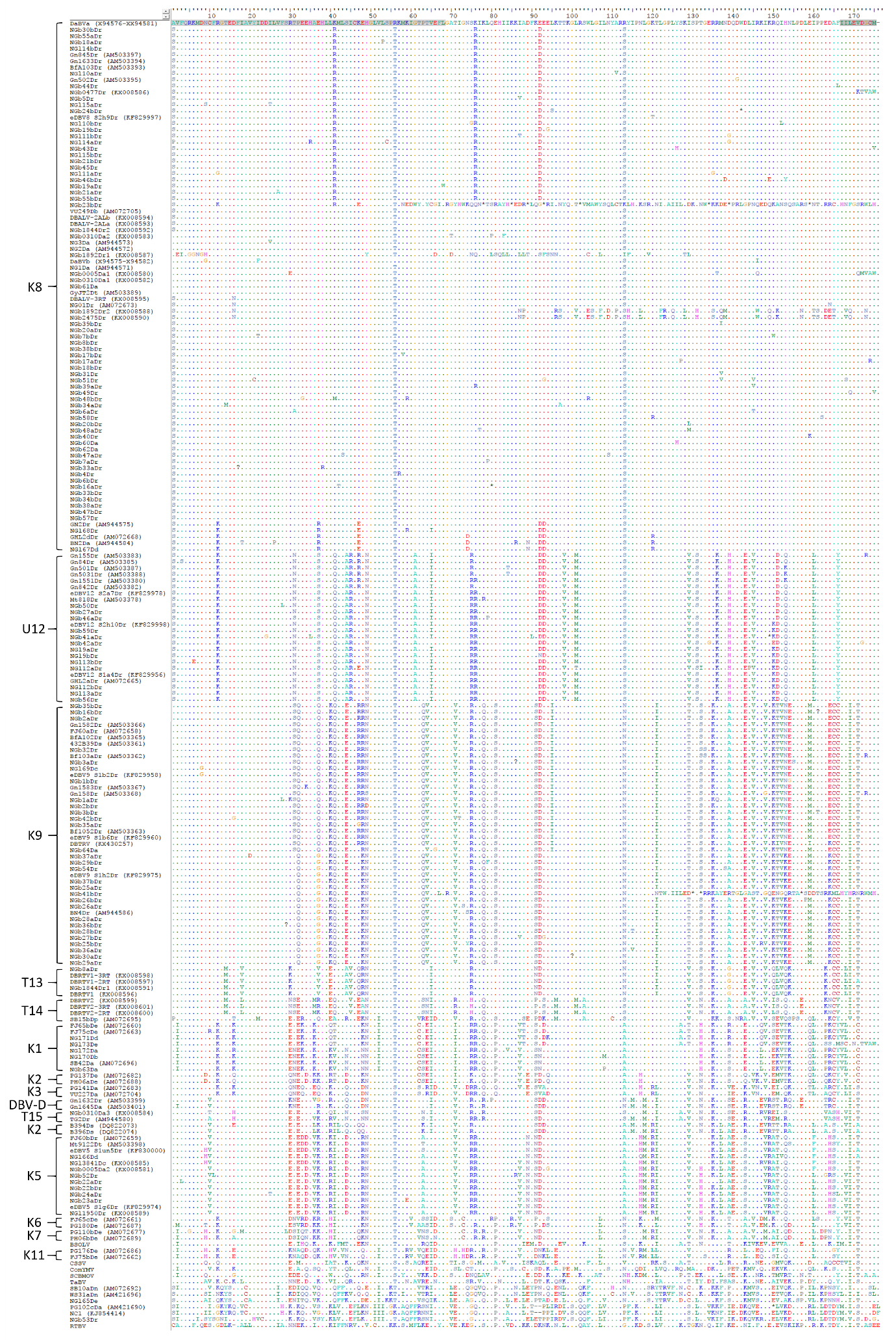
**

**Figure S2.** Protein alignment from deduced amino acid sequences of partial RT-RNaseH nucleotide sequences of 114 yam badnavirus sequences determined in this study together with other members of the family *Caulimoviridae*. Included in the analysis are partial RT-RNaseH sequences with names and accession numbers from GenBank of previously analysed yam samples by Bömer et al. [1], Bousalem et al. [2], Eni et al. [3], Kenyon et al. [4], Seal et al. [5] and Umber et al. [6,7]. Equivalent sequences from CSSV (AJ781003), BSOLV (AJ002234), ComYMV (NC001343), SCBMOV (M89923), TaBV (AF357836) and outgroup RTBV (X57924) were added, as well as representative sequences of all monophyletic groups described by Bousalem et al. [2], where DBV-D = Dioscorea bacilliform virus D, by Umber et al. [6] and by Kenyon et al. [4], denoted by U12 and K1–K11 respectively. Three novel monophyletic groups T13-15 described by Bömer et al. [1] were also included. Protein sequences were aligned using CLUSTAL OMEGA (<http://www.ebi.ac.uk/Tools/msa/clustalo/>) [8] and further processed in BioEdit version 7.2.5 [9]. All protein sequences analysed in this study were compared to the reference sequence of *Dioscorea bacilliform alata virus* (DBALV or DaBVa, X94576-XX94581, [10]). The C-terminal part of the RT domain (position 1–68) and the beginning of the RNaseH domain (position 167–175) within the partial RT-RNaseH sequence of DaBVa are highlighted (grey boxes).

**References**

1. Bömer, M.; Turaki, A.; Silva, G.; Kumar, P.; Seal, S. A Sequence-Independent Strategy for Amplification and Characterisation of Episomal Badnavirus Sequences Reveals Three Previously Uncharacterised Yam Badnaviruses. *Viruses* **2016**, *8*, 188.

2. Bousalem, M.; Durand, O.; Scarcelli, N.; Lebas, B. S. M.; Kenyon, L.; Marchand, J. L.; Lefort, F.; Seal, S. E. Dilemmas caused by endogenous pararetroviruses regarding the taxonomy and diagnosis of yam (*Dioscorea* spp.) badnaviruses: Analyses to support safe germplasm movement. *Arch. Virol.* **2009**, *154*, 297–314.

3. Eni, A. O.; Hughes, J. d’A; Asiedu, R.; Rey, M. E. C. Sequence diversity among badnavirus isolates infecting yam (*Dioscorea* spp.) in Ghana, Togo, Benin and Nigeria. *Arch. Virol.* **2008**, *153*, 2263–72.

4. Kenyon, L.; Lebas, B. S. M.; Seal, S. E. Yams (*Dioscorea* spp.) from the South Pacific Islands contain many novel badnaviruses: Implications for international movement of yam germplasm. *Arch. Virol.* **2008**, *153*, 877–889.

5. Seal, S.; Turaki, A.; Muller, E.; Kumar, P. L.; Kenyon, L.; Filloux, D.; Galzi, S.; Lopez-Montes, A.; Iskra-Caruana, M. L. The prevalence of badnaviruses in West African yams (*Dioscorea cayenensis-rotundata*) and evidence of endogenous pararetrovirus sequences in their genomes. *Virus Res* **2014**, *186*, 144–154.

6. Umber, M.; Filloux, D.; Muller, E.; Laboureau, N.; Galzi, S.; Roumagnac, P.; Iskra-Caruana, M. L.; Pavis, C.; Teycheney, P. Y.; Seal, S. E. The genome of African yam (*Dioscorea cayenensis-rotundata* complex) hosts endogenous sequences from four distinct badnavirus species. *Mol. Plant Pathol.* **2014**, *15*, 790–801.

7. Umber, M.; Gomez, R.-M.; Gélabale, S.; Bonheur, L.; Pavis, C.; Teycheney, P.-Y. The genome sequence of Dioscorea bacilliform TR virus, a member of the genus *Badnavirus* infecting *Dioscorea* spp., sheds light on the possible function of endogenous *Dioscorea* bacilliform viruses. *Arch. Virol.* **2017**, *162*, 517–521.

8. Sievers, F.; Wilm, A.; Dineen, D.; Gibson, T. J.; Karplus, K.; Li, W.; Lopez, R.; McWilliam, H.; Remmert, M.; Söding, J.; Thompson, J. D.; Higgins, D. G. Fast, scalable generation of high-quality protein multiple sequence alignments using Clustal Omega. *Mol. Syst. Biol.* **2011**, *7*, 539.

9. Hall, T. BioEdit: A user-friendly biological sequence alignment editor and analysis program for Windows 95/98/NT. *Nucleic Acids Symp. Ser.* 1999, *41*, 95–98.

10. Briddon, R. W.; Phillips, S.; Brunt, A.; Hull, R. Analysis of the sequence of *Dioscorea alata* bacilliform virus; comparison to other members of the badnavirus group. *Virus Genes* **1999**, *18*, 277–283.
